# Considerations for Germline Testing in Melanoma: Updates in Behavioral Change and Pancreatic Surveillance for Carriers of *CDKN2A* Pathogenic Variants

**DOI:** 10.3389/fonc.2022.837057

**Published:** 2022-03-16

**Authors:** Kristen Pauley, Ambreen Khan, Wendy Kohlmann, Joanne Jeter

**Affiliations:** ^1^ Family Cancer Assessment Clinic, Huntsman Cancer Institute, Salt Lake City, UT, United States; ^2^ Department of Internal Medicine, Huntsman Cancer Institute, Salt Lake City, UT, United States

**Keywords:** CDKN2A, melanoma, pancreatic cancer, surveillance, behavior change, hereditary cancer syndromes

## Abstract

The largest proportion of hereditary melanoma cases are due to pathogenic variants (PVs) in the *CDKN2A/p16* gene, which account for 20%-40% of familial melanomas and confer up to a 30%-70% lifetime risk for melanoma in individuals with these variants. In addition, PVs in the *CDKN2A* gene also increase risk for pancreatic cancer (~5–24% lifetime risk). Individuals with PVs in the *CDKN2A* gene also tend to have an earlier onset of cancer. Despite these known risks, uptake of germline testing has been limited in the past, largely due to perceptions of limited benefit for patients. Prevention recommendations have been developed for individuals with *CDKN2A* PVs as well the providers who care for them. On the patient level, behavioral modifications regarding melanoma prevention such as wearing sunscreen, limiting prolonged sun exposure and practicing general sun safety can help reduce risks. Germline testing can provide motivation for some individuals to adhere to these lifestyle changes. On the provider level, pancreatic cancer surveillance for individuals with *CDKN2A* PVs has been increasingly endorsed by expert consensus, although the efficacy of these surveillance methods remains under study. This review summarizes the updated surveillance guidelines for individuals with *CDKN2A* PVs and explores the impact of genetic counseling and testing in influencing behavioral changes in these individuals.

## Background

Most melanomas are sporadic; however, between 7-15% of melanomas occur in those with a family history of the cancer (1, 2). Many factors are involved in increasing an individual’s risk for melanoma, most of which influence a family as a whole. While sun exposure experiences, skin pigmentation, and geographic location have been well-characterized as risk factors (3–8), more recently, genetics have been a topic of interest in the melanoma world. Germline pathogenic variants (PVs) in a number of genes predispose to melanoma (9–12), but the largest proportion of familial melanoma cases (20%-40%) are due to PVs in the gene *CDKN2A (cyclin-dependent kinase inhibitor 2A)* (2, 12).

*CDKN2A* functions as a tumor suppressor gene, and somatic *CDKN2A* PVs are commonly found in both sporadic and hereditary melanomas (1). This gene is located on chromosome 9p21.3 and has two main transcripts (isoforms 1 and 4). Isoform 1 encodes the protein p16 (INK4a), while isoform 4 encodes the protein p14 (ARF). Germline PVs in the *CDKN2A* gene more commonly affect protein p16 than p14 and typically affect function of the G1/S checkpoint in the cell cycle by inhibiting cyclin-dependent kinases CDK4 and CDK6. This inhibition allows for uncontrolled cellular proliferation, which has many downstream carcinogenic affects (13). For simplicity, we will be using *CDKN2A* to refer to the PVs that occur in the p16 isoform as these are more common and better described than PVs in the p14 isoform.

## Familial Atypical Multiple Mole Melanoma Syndrome

Germline PVs in the *CDKN2A* gene are consistent with the condition called familial atypical multiple mole melanoma syndrome (FAMMM) (13). FAMMM is an autosomal dominant condition characterized by a large number of melanocytic nevi (often >50), up to 65-fold increased risk for cutaneous melanoma, and a 13-47-fold increased risk for pancreatic cancer (13–15). This translates to a 30%-70% lifetime risk for melanoma and a 5%-24% lifetime risk for pancreatic cancer (16, 17). In addition, other cancers have been observed in carriers, although actionable guidelines for increased surveillance for these cancers are not available at this time (18, 19).The penetrance rate for melanoma in individuals with *CDKN2A* PVs is estimated at 58-92% by age 80 (13, 20, 21). This variance in penetration may be related to location and associated sun exposure, although studies are conflicting on this point (22, 23). Variants in *MC1R*, often but not always associated with a red hair phenotype, can act as a modifier gene for *CDKN2A* mutation carriers, as well as their known effect as an independent low-penetrance susceptibility gene for melanoma (24). Histopathologic characteristics and somatic mutations of melanomas in individuals with *CDKN2A* PVs are similar to those with sporadic melanoma (13, 14, 25–28). Of note, several *CDKN2A* patients have been reported with melanomas with coexisting *BRAF* and *NRAS* mutations, which is uncommon in sporadic melanomas (28). Higher melanoma mortality rates have been described in *CDKN2A* families than in wild-type melanoma families (10); however other studies have found no difference in survival rates between *CDKN2A* carriers and non-carriers (29). (see **Table 1**)

**Table 1:** Summary of data on age of onset, penetrance and lifetime risks of FAMMM-associated cancer.

Cancer	Age of onset	Penetrance	Lifetime risks
Melanoma	30-45 years	58%-92% by age 80	30%-70% absolute risk, depending on other risk factors (family history, geographic location, others) (16, 17)
Pancreatic	58 years	Not well established One Dutch study quotes 17% penetrance by 75 years (30)	5%-24% absolute risk (16, 17) Relative risk = 43.8 95% CI = 13.8 – 139 (31)
Astrocytoma/Brain	Not well established	n/a	Relative risk = 1.9, 95% CI = 0.2 to 7.1, P = 0.58 (19)
Wilms tumor	Not well established	n/a	Relative risk = 40.4, 95% CI = 3.4 to 352.7, P = 0.005 (19)
Colon/GI	Not well established	n/a	Relative risk= 1.9 95% CI = 0.9 to 3.4, P = 0.10 (19)
Respiratory/lung	Not well established	n/a	Relative risk = 15.6 95% CI = 5.4 to 46 (31) Relative risk = 1.4, CI = 0.6 to 3.0, P = 0.44 (19)
Upper digestive	Not well established	n/a	Relative risk = 17.1 95% CI = 6.3 to 46.5 (31)

n/a, Not available.

Clinical characteristics of FAMMM include a large number of atypical melanocytic nevi; however, multiple nevi, while characteristic, are not diagnostic of FAMMM (1, 32). Multiple and/or dysplastic nevi are not restricted to inherited syndromes and are considered a strong risk factor for both sporadic melanoma and melanoma in *CDKN2A* carriers (33–36). Atypical nevi may transform into malignant melanoma, but melanomas in FAMMM patients often also develop on normal skin (13, 33, 37, 38).

Melanoma diagnosis in FAMMM cohorts typically occurs over a decade earlier than that in sporadic melanoma cases. Sporadic melanoma is typically diagnosed between the ages of 53-61, whereas individuals with a *CDKN2A* PV are often diagnosed between ages 30-45 (1, 39–41). The youngest reported cases of melanoma in FAMMM families have been seen at age 13 (42, 43). Additionally, individuals with *CDKN2A* PVs have an increased probability of multiple primary melanomas: one study reported a 23% incidence of second melanoma primary diagnosed within 5 years of the first, representing a 10-fold increase over that of melanoma patients without *CDKN2A* PVs (39). In a genotype-phenotype correlation study, multiple primary melanomas were the most predictive factor for presence of a CDKN2A mutation (44). Given the earlier presentation of melanoma in this population and potential for multiple primary lesions, increased and intensive surveillance for cutaneous melanomas is routinely recommended with screening starting at a young age (13, 45).

## Melanoma Surveillance

Many groups have provided recommendations for high-risk families with *CDKN2A* PVs, which include increased frequency of clinical skin examinations beginning in childhood (12, 46, 47). Suggested surveillance include clinical skin examinations yearly or biannually starting from age 10 with monthly self-examination of the skin beginning in childhood (47). When identified, suspicious moles should be biopsied and removed (47). Lifestyle modifications have been recommended for individuals with *CDKN2A* PVs that include limiting exposure to the sun and to ultraviolet radiation. Protective clothing should be worn when exposure is unavoidable (47). (see **Table 2**)

**Table 2:** Summary of surveillance recommendations for *CDKN2A* carriers.

**Cancer**	**Initiation**	**Methods**	**Interval**	**Additional information**
Melanoma	10 years	Self-examination	Monthly	Look for abnormalities in growth, shape, or coloring
		Clinical skin examination including: - Nevi - Scalp - Oral mucosa - Genitals (47)	Yearly or biannually	ABCDE Features: - Asymmetry - Border irregularity - Color variegation - Diameter >6mm - Evolution (48)
Pancreatic	40 years or 10 years earlier than earliest age of diagnosis in family	EUS and/or MRCP (49, 50)	Yearly if no abnormalities found	Should be performed at experienced high-volume centers (49)
		Fasting blood glucose and/or HbA1c (50)	Routine	
Astrocytoma/Brain, Wilms, Colon, Upper GI, Respiratory Tract	No consensus	No consensus	No consensus	

Full body skin examinations should include the scalp, oral mucosa, and genitals, as significant variability has been reported regarding location of melanomas (41). A healthcare provider should examine nevi for features of melanoma every 6 to 12 months. The patient should look for abnormalities in growth, shape or coloring through self-examinations of the skin. The ABCDE features (Asymmetry, Border irregularity, Color variegation, Diameter >6mm, Evolution) of melanoma should be screened for in these patients (13, 48).

## Behavioral Changes in *CDKN2A* Carriers

Predictive genetic testing has been shown to increase the uptake of cancer screening and prevention (51–53). In the past, concerns were raised about offering predictive DNA analysis for families suspicious of harboring a *CDKN2A* PV outside of defined research protocols. The concern was that the likelihood of finding a PV was low and the efficacy of melanoma prevention was lacking (54). In general meta-analysis of the benefits of predictive genetic testing for disease prevention in cohorts of multiple complex hereditary conditions showed mixed results (52, 55). In studies of *CDNK2A* carriers specifically, positive outcomes were reported one year post-counseling, including fewer reported sunburns and lower daily ultraviolet radiation dose compared to baseline analysis (53). Another study found that two years following genetic counseling, unaffected *CDKN2A* carriers reported improvements in following the recommendation to undergo annual total body skin examinations and increased thoroughness in their monthly skin self-examinations (51). Genetic counseling is recommended by the NCCN for familial melanoma following the “rule of three,” including three family members with melanoma/pancreatic cancer/astrocytoma on the same side of the family or an individual with three malignant melanomas or associated tumors (49).

Genetic testing to identify hereditary cancer risks has the potential for preventative surveillance and medical management options if a genetic predisposition is identified in an individual. While some hereditary cancer syndromes have surveillance guidelines that require routine medical follow-ups and options for additional imaging or surgery to reduce risks, hereditary predisposition to melanoma can be complicated by its multifactorial nature. The greater onus of prevention may fall on the individual. Behavioral modifications such as wearing sunscreen, limiting prolonged sun exposure and practicing general sun safety can allow some individuals to feel a greater sense of control in their medical management, but can also create limitations for other individuals who may not feel adequately prepared to make such behavioral modifications (56, 57).

Given the risk for melanoma is impacted by environmental factors such as UV exposure, a deeper understanding of the behavioral changes among individuals with a *CDKN2A* PV is important in tailoring medical management and targeting surveillance efforts. Several studies have indicated that identification of carrier status with a *CDKN2A* pathogenic variant can have cognitive and behavioral impacts beyond family history-based risk assessment alone (53, 58, 59). For example, one study showed that two years after undergoing genetic testing, *CDKN2A* carriers without a personal history of melanoma were found to have a 30% increase in adherence to total body skin examination (TBSEs) (p=0.032, one tailed) (60). This adherence was comparable to family members with melanoma who tested positive for the PV (p= 0.635).

While receiving a test result indicating a *CDKN2A* PV has been showing to have dramatic effects on behavior, not all patients undergoing testing will receive this result. Testing negative for a familial *CDKN2A* has not been found to have negative effects such as promoting increased UV exposure. At-risk relatives from melanoma-prone families without a known genetic etiology and for whom genetic testing was not available, also have been shown to benefit from genetic counseling about melanoma risk. Following genetic counseling they also exhibited significantly decreased UV exposure, though not as quickly or to the extent as *CDKN2A* carriers (53).

*CDKN2A* testing and test reporting in these studies was conducted in the setting of pre- and post-test genetic counseling. Pairing genetic testing with appropriate genetic counseling will maximize the benefit of this information for patients. Studies have shown that relatives at risk for *CDKN2A* PVs exhibit high levels of interest in genetic testing, similar to levels of interest in families with other hereditary cancer syndromes.

## Predictive Genetic Testing for Minors

Offering genetic testing to children is recommended only for conditions in which early intervention is available and the potential benefit of testing at that age outweighs the potential psychological harms (13). While the melanoma risk associated with *CDKN2A* PVs typically present in adulthood, screening recommendations initiate at age 10. Additionally, childhood is a time of significant UV exposure, and testing earlier in life may present an opportunity to minimize exposures that would contribute to melanoma risk later in life.

Genetic testing and counseling for *CDKN2A* has been shown to improve photoprotective behaviors among children (ages 10-15y), decrease sunburns by over 50% (p>.05), and increase adherence to sun-protective behaviors (55.6% vs. 88.9%, p = 0.04) one year after genetic counseling and testing (61). The decrease in sunburns and adherence to sun protection was reported equally by both carriers and non-carriers (p > 0.05) highlighting the importance of pre-test genetic counseling in improving awareness regarding sun-protective behaviors (61). There was no perceived increase noted in anxiety or depression among minors who underwent genetic testing for *CDKN2A*. Counseling and testing of children may heighten parents’ awareness of the need for sun protection in childhood.

It should be acknowledged that there are many barriers to sustainable and life-long behavioral changes and these can be challenging for individuals and families. According to Wu et al., peer influence can be an important factor impacting engagement in sun protective behaviors among children (56). Family modeling and communication, such as parents modeling preventative behaviors, can allow for improved engagement in sun protective behaviors among children. Interventions targeting education for broader populations regarding sun protection and dispelling of myths related to UV exposure/sun safety, such as the perception of reduced UVR exposure in winter, may be beneficial in addressing gaps in education and awareness among the general population.

## Pancreatic Cancer Surveillance

Pancreatic ductal adenocarcinoma (PDAC) is seen in association with FAMMM as the second most frequent cancer diagnosis in these kindreds (14, 15). Pancreatic cancer is often diagnosed at later stages, which is associated with poorer prognoses (62, 63). Less than ¼ of patients are candidates for potentially curative surgical resection at the time of diagnosis (64), therefore early detection is extremely important in improving survival outcomes (50).

While effective screening and prevention measures for melanoma exist, the efficacy of pancreatic cancer surveillance has not been as well established (65). It is also unknown how individuals with a *CDKN2A* PV may make behavioral changes regarding their pancreatic cancer risk, given there is greater individual control over melanoma prevention than pancreatic cancer prevention, at least at this time. In one study disclosing the return of research results, 85.7% (n = 12) of *CDKN2A* carriers indicated that they planned to have their pancreas checked in the next six months. However, not all carriers who intended to be screened for pancreatic cancer did so within six months. Those with positive *CDKN2A* results were more likely to communicate these results to their healthcare teams than non-carriers (66).

## PDAC Surveillance Guidelines

Pancreatic cancer screening guidelines have evolved over the years. Most recently, the International Cancer of the Pancreas Screening (CAPS) Consortium and the National Comprehensive Cancer Network (NCCN) have established consensus guidelines for surveillance of high-risk individuals (49, 50). Current CAPS and NCCN recommendations support pancreatic cancer screening for individuals with *CDKN2A* PVs, regardless of their family history.

For CDKN2A PV carriers, these guidelines recommend initiation of surveillance 10 years earlier than the earliest age of pancreatic cancer diagnosis in the family, or at age 40, whichever is earlier. The NCCN guidelines recommend that individuals considered to be at high risk for pancreatic cancer pursue these screenings at experienced high-volume centers after having in-depth discussions about the benefits and limitations of these screenings with their healthcare providers (49).

Surveillance methods include annual imaging with endoscopic ultrasound (EUS) and/or MRI/Magnetic resonance cholangiopancreatography (MRCP) per both CAPS and NCCN recommendations. CAPS guidelines recommend routine testing for late onset diabetes with fasting blood glucose and/or HbA1c, adding that high-risk individuals with new-onset diabetes should prompt additional screening (50). One year interval surveillance was recommended for those without abnormalities on imaging (49, 50). However, the CAPS Consortium did not reach a consensus on how to alternate the two screening modalities (50).

The Dutch Familial Pancreatic Cancer surveillance study performed a prospective study aimed at determining the long-term yield of PDAC surveillance in high-risk individuals between the years 2006-2019 (63). PDACs found through surveillance in the high-risk group were more likely to be resectable than sporadic PDACs diagnosed on the basis of development of symptoms. Of the 96 participants with CDKN2A PVs in this study, 7 were found to have PDAC through surveillance. EUS was found to be a superior imaging tool at detecting PDAC lesions with a solid component when compared to MRI/MRCP, while MRI/MRCP was found to be more sensitive at identifying small (sub-cm) cystic lesions. The diagnostic yield of PDAC was beneficial in high-risk patients, including those with *CDKN2A* PVs, but timely identification of disease in these patients still remains challenging. Individuals included in the study were highly adherent to scheduled procedures, which suggests that those with PDAC susceptibility PVs are ideally suited for increased surveillance (63).

Other studies have shown that PDAC surveillance of *CDKN2A* PV carriers is beneficial in detecting PDACs at a more resectable stage (66). Prospective screening data from three European centers were collected. Of those individuals who participated in surveillance programs diagnosed with PDAC, the resection rate was found to be 75% with a 5-year survival rate of 24% (compared historically to 13-21.2% with a 5-year survival rate of 4-7% for sporadic PDAC) (62, 66).

A second study following patients enrolled in the Cancer of the Pancreas Screening cohort also found strong evidence supporting the use of pancreatic surveillance in high-risk individuals (62, 67). This study found the majority of PDACs detected during screening to be resectable (90%) with a significantly increased 3-year survival outcome (85%). These two studies highlight the potential benefit of PDAC surveillance in high-risk cohorts and were used to justify the update to the CAPS guidelines (50).

## Conclusions

Historically germline genetic testing for cancer susceptibility was encouraged for genes with established clinical utility (68, 69). For many years, *CDKN2A* genetic testing has been felt to limit uptake on this basis. In recent years, developments in the behavioral science literature as well as the pancreatic surveillance literature have altered the risk-benefit ratio in *CDKN2A* testing. Behavioral literature has demonstrated increased sun-protective behaviors and surveillance, not just for individuals who were positive for *CDKN2A* PVs but also for those who underwent genetic evaluation for this condition. Additionally, pancreatic surveillance has been effective in identifying asymptomatic pancreatic cancers in this population and may be effective in down staging this disease. For this reason, expert consensus has recommended pancreatic cancer surveillance for all individuals with *CDKN2A* PVs (49, 50). Based on data emerging in these two areas, re-evaluation of the clinical utility of germline *CDKN2A* testing is appropriate.

## Author Contributions

AK, KP, WK, and JJ contributed to conception and design of the manuscript. AK and KP wrote the first draft of the manuscript. All authors contributed to manuscript revision, read, and approved the submitted version.

## Author Disclaimer

The content is solely the responsibility of the authors and does not necessarily represent the official views of the NIH.

## Conflict of Interest

The authors declare that the research was conducted in the absence of any commercial or financial relationships that could be construed as a potential conflict of interest.

## Publisher’s Note

All claims expressed in this article are solely those of the authors and do not necessarily represent those of their affiliated organizations, or those of the publisher, the editors and the reviewers. Any product that may be evaluated in this article, or claim that may be made by its manufacturer, is not guaranteed or endorsed by the publisher.
